# Is cognitive emotion regulation mediating effects of childhood maltreatment on suicidal ideation: a cross-sectional retrospective study

**DOI:** 10.3389/fpsyt.2025.1553687

**Published:** 2025-06-06

**Authors:** Mahtab Sabzehparvar, Sohrab Amiri

**Affiliations:** ^1^ Department of General Psychology, Faculty of Literature, Humanities and Social Sciences, Islamic Azad University, Tehran, Iran; ^2^ Spiritual Health Research Centre, Life Style Institute, Baqiyatallah University of Medical Sciences, Tehran, Iran

**Keywords:** emotion regulation, adolescent, suicidal ideation, childhood maltreatment, suicide

## Abstract

**Background:**

This research aims to explore the impact of childhood maltreatment and cognitive emotion regulation strategies on suicidal ideation among adolescents. Additionally, it examines the mediating role of emotion regulation strategies in influencing suicidal ideation.

**Methods:**

This cross-sectional study focused on adolescents aged 14 to 20 and involved participants selected through cluster sampling from secondary school levels, including both boys and girls. The research involved a sample size comprising 330 participants. The survey was conducted over a period from May 2024 to June 2024. The relationship between childhood maltreatment (exposure), cognitive emotion regulation (mediation), and suicidal ideation (outcome) was assessed using Pearson’s correlation coefficient. Additionally, structural equation modeling with maximum likelihood estimation was employed to examine the role of the mediator variable.

**Results:**

Childhood maltreatment and maladaptive cognitive emotion regulation were identified as predictors of suicidal ideation, with Beta coefficients of 0.28 and 0.33, respectively. On the other hand, adaptive cognitive emotion regulation served as a protective factor against suicidal ideation, reflected in a Beta coefficient of -0.25. Additionally, the indirect pathways from childhood maltreatment to suicidal ideation via maladaptive and adaptive cognitive emotion regulation revealed partial mediation effects.

**Conclusions:**

Emotion regulation plays a mediating role in the link between childhood maltreatment and suicidal ideation. However, the impact of childhood maltreatment remains a significant factor in influencing suicidal thoughts. Prioritizing the mental health of children within health policies and screening efforts is therefore essential.

## Introduction

Adolescence is one of the periods full of ups and downs in life, and the development of behavioral problems and psychopathology during this period is very complicated (1). Comorbidity of behavioral and psychopathological problems in adolescence is an important concern as these are associated with more severe disorders and increased suicide rates (2).

Suicide is one of the most important health issues (3) and has wide-ranging effects on individuals, families, societies, and countries, and its long-term effects remain (4). According to the World Health Organization (WHO), approximately 703,000 people die by suicide every year in the world, and a higher rate has also attempted suicide (4). In the latest published report, it is estimated that 759,028 people die by suicide in the world, the share of men is almost twice that of women, and it is equal to 523,883 for males and 235,145 for females (5). Age-standardized rates of mortality of suicide are 9 per 100,000, 12.6 for males, and 5.4 for females (5). Suicide occurs in all periods of life, and in the period between the ages of 15–29 years, it has the highest disease burden, so it is the fourth leading cause of death in this period (4). Suicide is the second leading cause of death for aged 10–24 years, the third-second leading cause in male adolescents aged 10–24 years, and is the most common cause of death among girls aged 15–19 years (6).

Suicidal ideation, which is often identified as suicidal thoughts or ideas, is a broad word to describe “contemplations, wishes, and preoccupations with death and suicide” (7). According to some studies, the prevalence of suicidal ideation has been reported as equal to 2.84% in the past week, 5.50% in the past year, and 18.49% during a lifetime (8). The prevalence of suicidal ideation in adolescents is higher than in the general population, the overall prevalence of suicidal ideation in adolescents is 16·9% (9). Girls had a higher prevalence of suicidal ideation than boys, 18·5% *vs* 15·1% respectively (9). The burden caused by suicide is high (10), and based on this, many factors related to suicide and suicidal ideation have been considered (8, 11, 12). One of the factors associated with suicidal ideation, planning, attempts, and suicide is childhood maltreatment (13–15).

The World Health Organization (WHO) defines childhood maltreatment as “ the abuse and neglect that occurs to children under 18 years of age”, including “physical and/or emotional ill-treatment, sexual abuse, neglect, negligence, and commercial or other exploitation, which results in actual or potential harm to the child’s health, survival, development or dignity in the context of a relationship of responsibility, trust or power” (16). Every year, 300 million children aged 2–4 are abused by their parents or caregivers through physical punishment or psychological violence (16). Childhood maltreatment is related to a range of mental disorders and physical diseases, as studies have shown that people who have been mistreated are exposed to various health problems, including cardiovascular disease (17), inflammation (18), obesity (19, 20), smoking (21, 22), depression (17), eating disorders (23) and personality disorders 24). Also, childhood maltreatment is known as an influencing factor on emotional regulation (25, 26). Exposure to childhood maltreatment may be associated with emotion dysregulation (27).

Cognitive emotion regulation (CER) is referred to as the “conscious, mental strategies individuals use to cope with the intake of emotionally arousing information” (28). Cognitive emotion regulation strategies can be classified into two main categories: adaptive strategies and maladaptive strategies (29). Adaptive and maladaptive cognitive emotion regulation strategies are related to a range of health-related issues (30, 31). Dysfunction in emotion regulation is known to be associated with self-injurious behaviors (32, 33). Among the emotion regulation strategies, some are protective against psychopathology and some are predictors of psychopathology (34). Based on this, it has been shown that people with self-injurious behaviors differ in the use of cognitive emotion regulation strategies (35). Therefore, different profiles in emotion regulation can be related to psychopathology profiles and self-injury and suicidal behaviors (35). It has been shown that people’s exposure to adverse experiences does not necessarily lead to difficulties in mental health (36), and it may be the difficulty in regulating emotions when facing stressful experiences that increases psychological vulnerability (37). What is important is that the effects of childhood maltreatment on suicidal ideation may be influenced by different factors, and since childhood maltreatment has a negative effect on a person’s emotional capacity, cognitive emotional regulation strategies may moderate the effects of childhood maltreatment. Therefore, the effects of adaptive and maladaptive cognitive-emotional regulation on the relationship between childhood maltreatment and suicidal ideation should be different, but it needs to be investigated.

Considering the role of childhood maltreatment in psychopathology in later life and also the role of adaptive and maladaptive emotion regulation strategies in increasing or decreasing vulnerability to mental health problems; the purpose of this research is to investigate the contribution of each of the dimensions of childhood maltreatment and cognitive emotion regulation strategies to suicidal ideation in adolescents. Our hypothesis was that adaptive and maladaptive emotion regulation strategies mediate the effects of childhood maltreatment on suicidal ideation.

## Method

### Study design and population

This research was a cross-sectional study that was conducted on adolescents aged 14-20. The target population was selected from secondary school levels using cluster sampling, which included both boys and girls. The study employed a cluster sampling method, utilizing the 22 districts of the city as the primary sampling frame. From this pool, five districts were randomly chosen, followed by the selection of two schools within each district. Subsequently, three classes were randomly drawn from each selected school to form the sample units. Finally, individual students from each class were randomly selected to participate in the study by completing the research questionnaires. The study population was surveyed from May 2024 to June 2024 and the data of this research was collected. To ensure the representativeness of the sample, the study population was selected more than the minimum required. Also, the gender ratio studied in this research was not equal and more of the population of girls was used.

### Sample size

G.power software (38) is used to measure the amount of sample required. Based on correlation PH 0, correlation PH1 0.2, α error probability 0.05, and power 0.95, the required sample size was determined to be 266, using G*power software (38). To be sure, the sample size is higher than the calculation formula was chosen and after collecting the data and cleaning up the data, finally 330 participants were evaluated in this research.

### Inclusion criteria

#1 The participants who were studying in high schools were eligible to participate in this research. #2 The age range of the participants for this research includes 14-20. #3 Both gender groups of boys and girls were eligible to participate in this study.

### Exclusion criteria

#1 Participants who had a history of chronic physical and psychological diseases were not eligible for this study. Individuals with a prior diagnosis of mental illness were excluded from the study. This subgroup represented a very small fraction, accounting for less than 2% of the overall sample. Their exclusion was primarily due to a lack of interest in participation expressed by both the individuals themselves and their families. Consequently, these cases were omitted prior to the commencement of the study. #2 Participants who had missing data more than 5% were excluded from the study.

### Study instruments

In the current research, two parts of data were collected, the first part included the demographic information of the research participants, and the second part included the measurement of the variables of exposure, mediator, and outcome in this research.

### #1Sociodemographic

A demographic questionnaire was created and used by researchers to collect information. This paper examined these items age, height, and weight to calculate BMI based on percentile (39), education level, employment status, family economic status, number of family members, history of disease, history of any drug use, who you live with?

### #2Beck scale for suicide ideation

The Beck scale for suicide ideation was developed in 1988 and contains 19 items that measure the intensity of suicidal wishes and suicide plans. The range of total scores of the questionnaire is 0 to 38, and higher scores indicate greater intensity of suicidal ideation (40). The first five items of this questionnaire are for screening, and if a person scores 0 in items 4 and 5, the questionnaire is complete and there is no need to continue answering. If a participant’s score in items 4 and 5 is > 0, all subsequent items must be answered (41). No cutoff point has been reported for this questionnaire (42). In the original version, favorable psychometric properties have been reported for this questionnaire (40), and this questionnaire has been used in different cultures and populations and has had favorable psychometric results (43–45). This questionnaire has been standardized in the Iranian population and shows desirable psychometric properties (43). Cronbach’s alpha coefficient was 0.837, also, factor analysis supported its factors (43).

### #3Childhood trauma questionnaire

The childhood trauma questionnaire is a self-report questionnaire that contains 28 items and is used to measure five dimensions of childhood trauma including sexual abuse, emotional abuse, physical abuse, emotional neglect, and physical neglect. Also, the three items in this questionnaire measure whether the participant intends to minimize problematic experiences within the family or not. The original version and long form of this questionnaire were made available in 1994 (46, 47), followed by its short version in 2003 (48). This questionnaire has been evaluated psychometrically in different populations and it shows its suitability in measuring childhood trauma (48–50). In the adolescent population, Cronbach’s alpha coefficient for each sub-scale is reported as follows Emotional abuse α = .89, Physical abuse α =.86, Sexual abuse α =.95, Emotional neglect α =.89, Physical neglect α =.78 (48). The scoring of this questionnaire is based on a 5-point Likert scale never true’ (1) to ‘very often true’ (5)(48). The version used in this study was in accordance with its Persian translation in Iran (51).

### #4Cognitive emotion regulation questionnaire-short form

A cognitive emotion regulation strategies questionnaire was formed in 1999 based on theoretical and experimental foundations(29). The short-form version of the original questionnaire has 18 items and like the original version, it includes 9 subscales “self-blame, Other-blame, Rumination, Catastrophizing, Putting intoPerspective, Positive Refocusing, Positive Reappraisal, Acceptance, and Planning”(52). Each of the subscales is measured based on two items. In total, it includes five subscales of adaptive cognitive emotion regulation strategies and four subscales of maladaptive cognitive emotion regulation strategies. Answering the items of this questionnaire is based on a 5-point Likert scale from 1 ((almost) never) to 5 ((almost). Higher scores indicate more use of the specific cognitive emotion regulation strategy. The score of each subscale is obtained from the sum of the components of that subscale and is between 2-10. The alpha coefficient of the subscales is between 0.68 and 0.81 (52). Also, other psychometric indicators indicate the desirability of this questionnaire (52). This questionnaire has been translated for the Iranian society and its standardization shows desirable psychometric properties (53).

### Statistical analysis

Descriptive indicators of the participants in this study were evaluated, including age, gender, family income, and employment. After scoring the scales related to childhood abuse, suicidal ideation, and cognitive emotion regulation, the descriptive index of each of these scales and their subscales was examined in the research population. Pearson’s correlation coefficient was used to evaluate the association between exposure (childhood maltreatment), mediation (cognitive emotion regulation), and outcome variables (suicidal ideation). Structural equation modeling with maximum likelihood was used to investigate the mediator variable. SPSS version-26 (IBM, USA) and AMOS-24 (54) were used.

## Results

A total of 330 adolescents were included in this study, of which 75% were boys and 25% were girls. The age of the participants was between 14–20 years (**Table 1**).

**Table 1:** Demographic descriptions of the participants.

Sociodemographic	n (%) n = 330
Sex	
Female	247 (74.8%)
Male	83 (25.2%)
Age (yrs.)	
14-15	41 (12.4%)
16-17	189 (57.3%)
18-20	100 (30.3%)
Employment status	
Working	72 (22.2%)
Not working	253 (77.8%)
Economic status	
Poor	21 (6.4%)
Moderate	238 (72.6%)
Good	69 (21%)
Father educational level	
Illiterate	22 (6.8%)
Diploma	202 (62.2%)
Bachelor	54 (16.6%)
M.sc and above	47 (14.4%)
Education Mother	
Illiterate	41 (12.4%)
Diploma	200 (60.6%)
Bachelor	65 (19.7%)
M.sc and above	24 (7.3%)
History of disease	
Yes	140 (42.4%)
No	190 (57.6%)
Material consumption	
Yes	67 (21%)
No	251 (79%)

**Table 2** shows the mean, standard deviation, and other descriptive indicators of childhood maltreatment, adaptive and maladaptive cognitive emotion regulation, and suicidal ideation.

**Table 2:** Descriptive statistics for childhood maltreatment, adaptive, and maladaptive cognitive emotion regulation, and suicidal ideation (N=330).

Variables	Minimum	Maximum	Mean	Std. deviation	Variance
Suicidal ideation	.00	2.00	.3945	.48825	.238
Maladaptive emotion regulation	1.00	4.63	2.8022	.72440	.525
Adaptive emotion regulation	1.40	6.20	3.2263	.80470	.648
Self-blame	2.00	10.00	5.2186	2.15917	4.662
Acceptance	2.00	10.00	5.9394	2.33304	5.443
Rumination	2.00	10.00	7.0547	2.14356	4.595
Positive Refocusing	2.00	13.00	5.9479	2.39308	5.727
Refocus on Planning	2.00	29.00	7.0488	2.48231	6.162
Positive reappraisal	2.00	10.00	6.9556	2.27185	5.161
Putting into perspective	2.00	10.00	6.3709	2.07383	4.301
Catastrophizing	2.00	10.00	5.6913	2.28196	5.207
Other-blame	2.00	10.00	4.4531	1.89769	3.601
Childhood maltreatment	32.32	104.00	46.1771	10.96496	120.230
Emotional abuse	5.00	22.00	7.2074	3.26207	10.641
Physical abuse	5.00	25.00	5.9581	2.40511	5.785
Sexual abuse	5.00	25.00	6.1409	2.97403	8.845
Emotional neglect	5.00	24.00	9.5462	4.93160	24.321
Physical neglect	5.00	21.00	6.9148	2.84292	8.082

### Pearson’s correlation analysis

Pearson’s correlation was used in the analysis of relationships. The correlation results between childhood maltreatment, cognitive emotion regulation, and suicidal ideation are shown in **Table 3**.

**Table 3:** Pearson’s correlation coefficient between childhood maltreatment, adaptive and maladaptive cognitive emotion regulation, and suicidal ideation.

Variables	1	2	3	4
Suicidal ideation	1			
Maladaptive emotion regulation	.307^**^	1		
Adaptive emotion regulation	-.231^**^	.122^*^	1	
Childhood maltreatment	.367^**^	.242^**^	-.188^**^	1

**. 0.01 *. 0.05.

Correlation results indicated that childhood maltreatment had a positive association with suicidal ideation (r=0.367; *P*<0.01). Based on this, as childhood maltreatment increases, suicidal ideation also increases.

Correlation results indicated that maladaptive emotion regulation had a positive association with suicidal ideation (r=0.307; *P*<0.01). Based on this, as the maladaptive cognitive regulation strategies increase, suicidal ideation also increases.

Correlation results indicated that adaptive emotion regulation had a negative association with suicidal ideation (r=-0.231; *P*<0.01). Based on this, as the adaptive cognitive regulation strategies increase, suicidal ideation decreases.

Correlation results indicated that childhood maltreatment had a positive association with maladaptive emotion regulation (r=0.242; *P*<0.01). Based on this, as childhood maltreatment increases, maladaptive emotion regulation also increases.

Correlation results indicated that childhood maltreatment had a negative association with adaptive emotion regulation (r=-0.188; *P*<0.01). Based on this, as childhood maltreatment increases, adaptive emotion regulation decreases.

**Supplementary Tables 1**-**3** show the correlation between subscales for childhood maltreatment, cognitive emotion regulation strategies, and suicidal ideation.

### Mediation analysis

**Figure 1:**
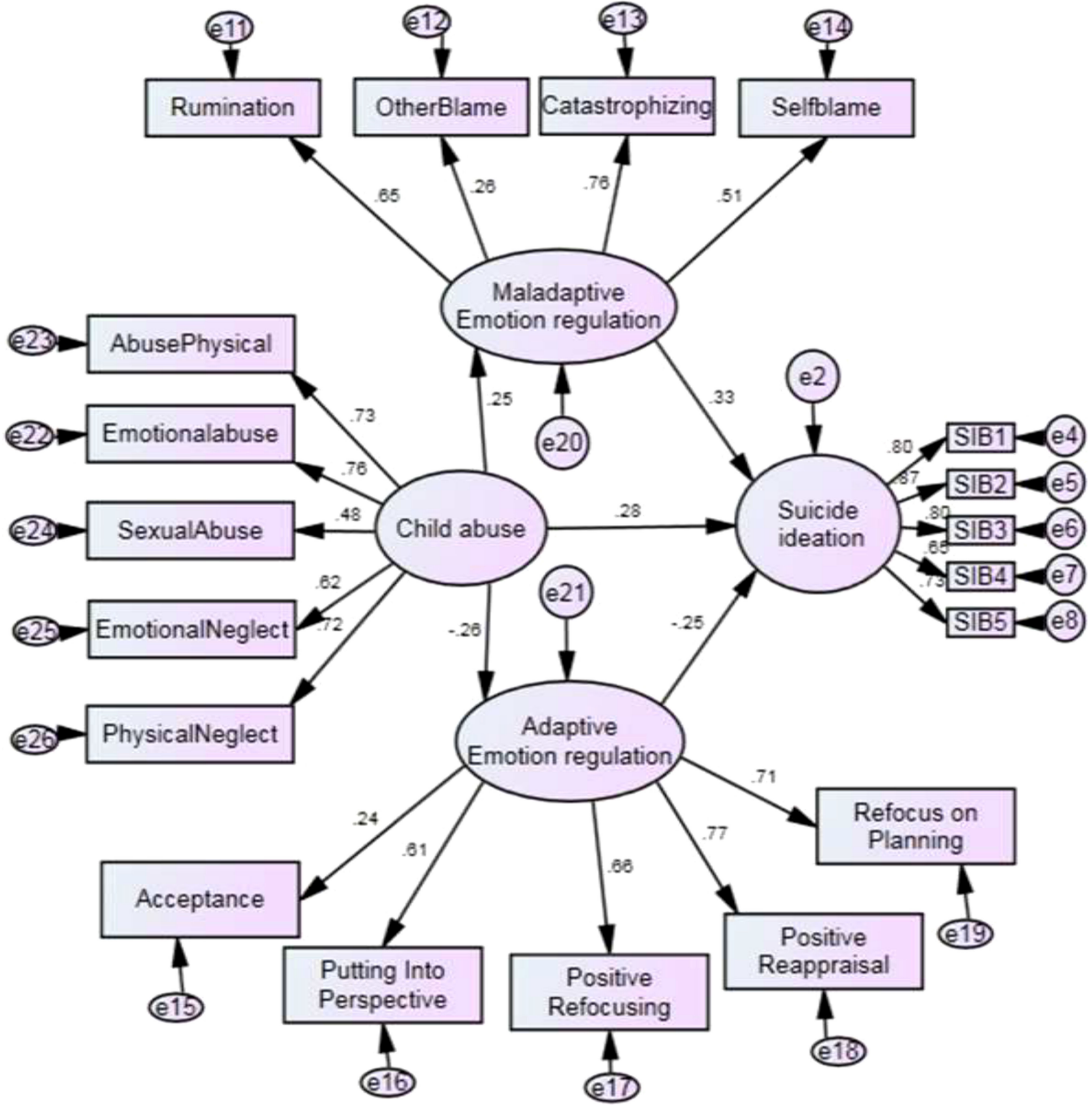
Childhood maltreatment affects suicidal ideation by mediating cognitive emotion regulation SIB. These refer to suicidal ideation items taken from the Beck Scale for Suicide Ideation.

**Figure 1** shows Beta coefficients between the exposure variable (childhood maltreatment) the mediating variable (cognitive emotion regulation) and the outcome variable (suicidal ideation).

The direct path coefficients between the exposure variable, mediator variable, and outcome variable are listed in **Table 4**.

**Table 4:** Direct effects between variables childhood maltreatment, adaptive, and maladaptive cognitive emotion regulation, and suicidal ideation.

Parameter	Estimate	Lower	Upper	P value
Childhood maltreatment to Maladaptive Emotion regulation	.254	.089	.390	.005
Childhood maltreatment to Adaptive Emotion regulation	-.263	-.405	-.116	.001
Maladaptive Emotion regulation to suicide ideation	.328	.203	.447	.000
Adaptive Emotion regulation to suicide ideation	-.249	-.363	-.121	.001
Childhood maltreatment to suicide ideation	.285	.136	.430	.001

Direct Beta coefficients from childhood maltreatment to suicidal ideation were 0.28 with a 95% confidence interval (0.13-0.43; P=0.001). It shows that childhood maltreatment is a significant predictor of suicidal ideation.

Direct Beta coefficients from childhood maltreatment to maladaptive cognitive emotion regulation was 0.25 with a 95% confidence interval (0.8-0.39; P=0.005). It shows that childhood maltreatment is a significant predictor for the increase of maladaptive cognitive emotion regulation.

Direct Beta coefficients from childhood maltreatment to adaptive cognitive emotion regulation was -0.26 with a 95% confidence interval (-0.40;-0.116; P=0.001). It shows that childhood maltreatment is a significant predictor for the decrease of adaptive cognitive emotion regulation.

Direct Beta coefficients from maladaptive cognitive emotion regulation to suicidal ideation was 0.32 with a 95% confidence interval (0.20;0.44; P<0.001). It shows that maladaptive cognitive emotion regulation is a significant predictor of suicidal ideation.

Direct Beta coefficients from adaptive cognitive emotion regulation to suicidal ideation were -0.24 with a 95% confidence interval (-0.367;-0.12; P<0.001). It shows that adaptive cognitive emotion regulation is a significant predictor for the decrease of suicidal ideation.

The indirect path coefficients between the exposure variable and outcome variable based on mediator variables are listed in **Table 5**.

**Table 5:** Indirect effects between variables.

Parameter	Estimate	Lower	Upper	P
Childhood maltreatment to suicide ideation via Maladaptive Emotion regulation	.022	.008	.044	.003
Childhood maltreatment to suicide ideation via Adaptive Emotion regulation	.017	.006	.034	.000

Indirect path coefficients from childhood maltreatment to suicide ideation via maladaptive cognitive emotion regulation was 0.02 with a 95% confidence interval (0.008;0.044; P<0.001). When the mediator variable entered into the model, the path coefficients coefficient was 0.02, but it was still significant. This mediation type is called “partial mediation”.

Indirect path coefficients from childhood maltreatment to suicide ideation via adaptive cognitive emotion regulation was 0.017 with a 95% confidence interval (0.006;0.033; P<0.001). When the mediator variable entered into the model, the path coefficients coefficient was 0.017, but it was still significant. This mediation type is called “partial mediation”.

The statistics related to the fit of the model are listed in **Table 6**. Chi-square/DF ≤ 5 indicates a reasonable fit (55). RMSEA ranging from 0.05 to 0.08 are considered acceptable (56).

**Table 6:** Model fit statistics.

Chi-square	Chi-square/DF	GFI	AGFI	CFI	RMSEA	RMR
472.275	3.21	0.87	0.83	0.85	0.08	0.42

Adjusted Goodness of Fit Index; CFI, Comparative Fit Index; RMSEA, Root Mean Square Error of Approximation; PMR, Root Mean Squared Residual; SRMR, Standardized Root Mean Squared Residual; CN, Critical N.

## Discussion

This research was conducted as a cross-sectional and retrospective study to examine the effects of childhood maltreatment and adaptive and maladaptive cognitive emotion regulation strategies on suicidal ideation in adolescents. Also, the other goal of this study, which was more important, was to investigate the mediating effects of adaptive and maladaptive cognitive emotion regulation strategies in the relationship between childhood maltreatment and suicidal ideation.

The investigation related to childhood maltreatment with the prediction of suicidal ideation based on structural equation modeling showed that childhood maltreatment was an important predictor of the risk of suicidal ideation in the studied adolescent population. This finding is consistent with previous studies that have shown that childhood maltreatment is associated with suicide (15, 57, 58). Some mechanisms have been pointed out in the relationship between childhood maltreatment and suicidal ideation and explained how childhood maltreatment affects (58). The experience of childhood maltreatment leads to a disturbance in the hypothalamic-pituitary-adrenal axis. For example, abused women have been shown to have lower levels of plasma cortisol compared to healthy women (59). This low level of cortisol has also been observed in people who have attempted suicide (60). On the other hand, the effects of childhood maltreatment on suicidal ideations can be through other mental health problems such as mentalization and depression (15). It has been proposed that the link between childhood maltreatment and suicidal ideation could be influenced by other significant factors (61). Addressing these mediators is essential for designing effective mental health interventions aimed at reducing the risk of suicide. The developmental model of mentalization (Peter 62), posits that the dynamic interaction between a parent and child lays the groundwork for future social relationships. Within this framework, the caregiver’s capacity to engage in mentalizing processes is pivotal in shaping the child’s attachment security. For individuals who have experienced childhood maltreatment, their opportunities for engaging in constructive emotional dialogue may have been constrained or marked by adverse emotional reactions from caregivers. Such experiences can impede the maturation of mentalizing capacities (15, 63), subsequently manifesting as broader challenges in social cognitive development, negative self-perceptions, and impaired capacity for interpersonal trust (64).

In the study of cognitive emotion regulation strategies, it was shown that adaptive emotion regulation is associated with a decrease in suicidal ideation, but on the contrary, maladaptive emotion cognitive regulation strategies are associated with an increase in suicidal ideation. Previous studies also show the role of maladaptive emotional regulation strategies in psychopathology (65, 66). The findings obtained from this research showed that the effects of childhood maltreatment on suicidal ideations were mediated through cognitive emotion regulation strategies. The mechanism through which cognitive emotion regulation strategies can mediate the effects of childhood maltreatment on suicidal ideation has been investigated in studies. Childhood maltreatment leads to impairment in cognitive functions (67, 68) and it also disrupts emotional regulation (69). This cognitive impairment can be directly related to suicide or indirectly by increasing vulnerability and suffering from other mental disorders (70–72). Growing evidence suggests that cognitive deficits and impairments could play a role in suicidality (73). The findings of our study could hold significant clinical implications, particularly in the context of Iran, where research in this domain is still in its nascent stages but is urgently needed to guide policy, identification, and prevention efforts. For example, psychotherapeutic interventions designed to prevent suicidal behaviors might gain added effectiveness by incorporating a focus on improving cognitive executive function and cognitive emotion regulation. This is especially relevant since deficits and disruptions in these areas are potentially modifiable (74).

This study is a retrospective cross-sectional study and causal relationships cannot be extracted. The results of mediation analysis support the hypothesis that emotion regulation partially mediates the relationship between childhood maltreatment and suicidal ideation. However, the effect sizes of the mediation paths are relatively small (indirect effects of 0.022 and 0.017), which raises questions about the practical significance of these findings. In addition to the variables studied in this research, the role of other emotional components in connection with suicidal ideation should be investigated. The study does not account for other potential confounding variables, such as comorbid mental health disorders (e.g., depression, anxiety), family dynamics, or socioeconomic status, which could also influence suicidal ideation. Our aim was to maintain a balanced gender ratio; however, due to insufficient cooperation from boys’ schools, this study was conducted solely on girls. In interpreting the gender differences observed in this study, it is important to consider the imbalance in the proportion of girls to boys. Suicidal behaviors, as defined theoretically, encompass two primary dimensions: suicidal ideation and suicide attempts. The present study, however, focused specifically on the assessment of suicidal ideation. This study examined suicidal ideation, and therefore caution is needed in generalizing its findings to other dimensions of suicide. The reliance on self-reported measures for childhood maltreatment, emotion regulation, and suicidal ideation introduces the potential for recall bias and social desirability bias. Adolescents may underreport or over report their experiences, which could affect the validity of the results.

### Clinical implications

Adolescence is a critical phase of development marked by significant emotional, personality, and physiological transformations, which can make young individuals more vulnerable to harm. During this time, mental health can be improved through education, early diagnosis, prevention, and timely treatment. As such, it is essential for health policies to prioritize monitoring psychological changes and addressing psychological trauma in adolescents, both on an individual level and within the broader public sphere.

## Conclusion

Childhood maltreatment and maladaptive emotion regulation were strong predictors of suicidal ideation, and adaptive emotion regulation was an inhibitor of suicidal ideation. Emotion regulation is a mediator in the relationship between childhood maltreatment and suicidal ideation, but the effects of childhood maltreatment are still determinants of suicidal ideation. It is necessary to pay much attention to the importance of mental health in childhood in health policy and screening so that a safe period can be passed and this safety will affect the entire life period.

## Data Availability

The raw data supporting the conclusions of this article will be made available by the authors, without undue reservation.
