# Supplementary material for: Is cognitive emotion regulation mediating effects of childhood maltreatment on suicidal ideation: a cross-sectional retrospective study
Source: Front Psychiatry. 2025 Jun 6;16:1553687. doi: 10.3389/fpsyt.2025.1553687 (PMC12179175; doi:10.3389/fpsyt.2025.1553687)
Supplement: Supplementary file 1 [file Table1.docx]

| **Supplementary Table 1. Pearson's correlation coefficient between emotion regulation and suicidal ideation** | | | | | | | | | | | | |
| --- | --- | --- | --- | --- | --- | --- | --- | --- | --- | --- | --- | --- |
|  | 1 | 2 | 3 | 4 | 5 | 6 | 7 | 8 | 9 | 10 | 11 | 12 |
| suicidal ideation | 1 |  |  |  |  |  |  |  |  |  |  |  |
| Maladaptive emotion regulation | .307^**^ | 1 |  |  |  |  |  |  |  |  |  |  |
| Adaptive emotion regulation | -.231^**^ | .122^*^ | 1 |  |  |  |  |  |  |  |  |  |
| Self-blame | .286^**^ | .662^**^ | .094 | 1 |  |  |  |  |  |  |  |  |
| Acceptance | .048 | .361^**^ | .493^**^ | .320^**^ | 1 |  |  |  |  |  |  |  |
| Rumination | .210^**^ | .739^**^ | .208^**^ | .348^**^ | .389^**^ | 1 |  |  |  |  |  |  |
| Positive refocusing | -.263^**^ | -.087 | .732^**^ | .011 | .123^*^ | -.074 | 1 |  |  |  |  |  |
| Refocus on planning | -.185^**^ | .031 | .766^**^ | -.016 | .178^**^ | .143^**^ | .428^**^ | 1 |  |  |  |  |
| Positive reappraisal | -.275^**^ | -.005 | .768^**^ | .008 | .178^**^ | .121^*^ | .507^**^ | .576^**^ | 1 |  |  |  |
| Putting Into Perspective | -.126^*^ | .137^*^ | .723^**^ | .004 | .237^**^ | .150^**^ | .480^**^ | .450^**^ | .410^**^ | 1 |  |  |
| Catastrophizing | .284^**^ | .802^**^ | -.014 | .362^**^ | .153^**^ | .501^**^ | -.189^**^ | -.005 | -.095 | .100 | 1 |  |
| Other-Blame | .035 | .501^**^ | .049 | .054 | .115^*^ | .130^*^ | .034 | -.043 | -.047 | .123^*^ | .268^**^ | 1 |
| **. 0.01 *. 0.05 | | | | | | | | | | | | |
|  | | | | | | | | | | | | |
