# Supplementary material for: Is cognitive emotion regulation mediating effects of childhood maltreatment on suicidal ideation: a cross-sectional retrospective study
Source: Front Psychiatry. 2025 Jun 6;16:1553687. doi: 10.3389/fpsyt.2025.1553687 (PMC12179175; doi:10.3389/fpsyt.2025.1553687)
Supplement: Supplementary file 2 [file Table2.docx]

| **Supplementary Table 2. Pearson's correlation coefficient between childhood maltreatment and suicidal ideation** | | | | | | | |
| --- | --- | --- | --- | --- | --- | --- | --- |
|  | 1 | 2 | 3 | 4 | 5 | 6 | 7 |
| suicidal ideation | 1 |  |  |  |  |  |  |
| Childhood Trauma | .367^**^ | 1 |  |  |  |  |  |
| Emotional abuse | .339^**^ | .783^**^ | 1 |  |  |  |  |
| Physical Abuse | .281^**^ | .731^**^ | .612^**^ | 1 |  |  |  |
| Sexual Abuse | .218^**^ | .615^**^ | .346^**^ | .419^**^ | 1 |  |  |
| Emotional Neglect | .327^**^ | .714^**^ | .470^**^ | .328^**^ | .205^**^ | 1 |  |
| Physical Neglect | .234^**^ | .786^**^ | .484^**^ | .514^**^ | .345^**^ | .593^**^ | 1 |
| **. 0.01 *. 0.05 | | | | | | | |
