# Supplementary material for: Is cognitive emotion regulation mediating effects of childhood maltreatment on suicidal ideation: a cross-sectional retrospective study
Source: Front Psychiatry. 2025 Jun 6;16:1553687. doi: 10.3389/fpsyt.2025.1553687 (PMC12179175; doi:10.3389/fpsyt.2025.1553687)
Supplement: Supplementary file 3 [file Table3.docx]

|  |  |  |  |  |  |  |  |  |  |  |  |  |  |  |  |  |  |  |  |
| --- | --- | --- | --- | --- | --- | --- | --- | --- | --- | --- | --- | --- | --- | --- | --- | --- | --- | --- | --- |
| **Supplementary Table 3. Pearson's correlation coefficient between childhood maltreatment and emotion regulation** | | | | | | | | | | | | | | | | | | | |
|  | 1 | | 2 | | 3 | 4 | 5 | 6 | 7 | 8 | 9 | 10 | 11 | 12 | 13 | 14 | 15 | 16 | 17 |
| Childhood Trauma | 1 | |  | |  |  |  |  |  |  |  |  |  |  |  |  |  |  |  |
| Emotional abuse |  | .783^**^ | | 1 |  |  |  |  |  |  |  |  |  |  |  |  |  |  |  |
| Physical Abuse |  | .731^**^ | | .612^**^ | 1 |  |  |  |  |  |  |  |  |  |  |  |  |  |  |
| Sexual Abuse |  | .615^**^ | | .346^**^ | .419^**^ | 1 |  |  |  |  |  |  |  |  |  |  |  |  |  |
| Emotional Neglect |  | .714^**^ | | .470^**^ | .328^**^ | .205^**^ | 1 |  |  |  |  |  |  |  |  |  |  |  |  |
| Physical Neglect |  | .786^**^ | | .484^**^ | .514^**^ | .345^**^ | .593^**^ | 1 |  |  |  |  |  |  |  |  |  |  |  |
| Maladaptive emotion regulation |  | .242^**^ | | .195^**^ | .167^**^ | .186^**^ | .210^**^ | .134^*^ | 1 |  |  |  |  |  |  |  |  |  |  |
| Adaptive emotion regulation |  | -.188^**^ | | -.090 | -.095 | -.162^**^ | -.166^**^ | -.144^**^ | .122^*^ | 1 |  |  |  |  |  |  |  |  |  |
| Self-blame |  | .157^**^ | | .169^**^ | .042 | .109^*^ | .161^**^ | .042 | .662^**^ | .094 | 1 |  |  |  |  |  |  |  |  |
| Acceptance |  | .072 | | .059 | .105 | .012 | .055 | .076 | .361^**^ | .493^**^ | .320^**^ | 1 |  |  |  |  |  |  |  |
| Rumination |  | .126^*^ | | .112^*^ | .131^*^ | .121^*^ | .128^*^ | .038 | .739^**^ | .208^**^ | .348^**^ | .389^**^ | 1 |  |  |  |  |  |  |
| Positive Refocusing |  | -.171^**^ | | -.073 | -.089 | -.177^**^ | -.168^**^ | -.103 | -.087 | .732^**^ | .011 | .123^*^ | -.074 | 1 |  |  |  |  |  |
| Refocus on Planning |  | -.129^*^ | | .001 | -.049 | -.087 | -.161^**^ | -.142^**^ | .031 | .766^**^ | -.016 | .178^**^ | .143^**^ | .428^**^ | 1 |  |  |  |  |
| Positive reappraisal |  | -.290^**^ | | -.200^**^ | -.196^**^ | -.234^**^ | -.185^**^ | -.243^**^ | -.005 | .768^**^ | .008 | .178^**^ | .121^*^ | .507^**^ | .576^**^ | 1 |  |  |  |
| Putting Into Perspective |  | -.139^*^ | | -.115^*^ | -.113^*^ | -.079 | -.118^*^ | -.091 | .137^*^ | .723^**^ | .004 | .237^**^ | .150^**^ | .480^**^ | .450^**^ | .410^**^ | 1 |  |  |
| Catastrophizing |  | .178^**^ | | .141^*^ | .100 | .131^*^ | .164^**^ | .095 | .802^**^ | -.014 | .362^**^ | .153^**^ | .501^**^ | -.189^**^ | -.005 | -.095 | .100 | 1 |  |
| Other Blame |  | .204^**^ | | .108 | .193^**^ | .148^**^ | .116^*^ | .204^**^ | .501^**^ | .049 | .054 | .115^*^ | .130^*^ | .034 | -.043 | -.047 | .123^*^ | .268^**^ | 1 |
| **. 0.01 *. 0.05 |  |  |  |  |  |  |  |  |  |  |  |  |  |  |  |  |  |  |  |
|  |  |  |  |  |  |  |  |  |  |  |  |  |  |  |  |  |  |  |  |
